# Human diseases caused by homozygous PTH1R mutations

**DOI:** 10.3389/fendo.2025.1641292

**Published:** 2025-08-19

**Authors:** Ignacio Portales-Castillo, Jakob Höppner, Harald Jüppner, Thomas J. Gardella

**Affiliations:** ^1^ Department of Medicine, Division of Nephrology, Washington University in St. Louis, St. Louis, MO, United States; ^2^ Endocrine Unit, Massachusetts General Hospital, and Harvard Medical School, Boston, MA, United States; ^3^ Pediatric Nephrology Unit, Massachusetts General Hospital, and Harvard Medical School, Boston, MA, United States

**Keywords:** PTH1R, Blomstrand, Eiken, tooth eruption, growth plate, delayed ossification, βarrestin, GPCR

## Abstract

The parathyroid hormone receptor type 1 (PTH1R) is a G protein-coupled receptor that mediates the actions of parathyroid hormone (PTH) in the regulation of blood calcium levels, as well as PTH-related protein (PTHrP) in the regulation of skeletal development. Severe loss-of-function homozygous mutations in PTH1R are incompatible with life as in Blomstrand’s lethal chondrodysplasia, characterized by accelerated growth plate ossification. More recently, homozygous mutations located in the transmembrane helices, extracellular domains and C-tail of the PTH1R were identified in patients with milder conditions characterized by variable degrees of skeletal and mineral abnormalities. These include delayed ossification in Eiken syndrome, hypocalcemia in a pseudohypoparathyroidism-like disorder, and non-syndromic primary failure of tooth eruption; which is usually caused by heterozygous PTH1R mutations. Recent detailed pharmacologic characterization of these PTH1R mutants has revealed new insights into how even subtle perturbations in PTH1R function can result in disease.

## Overview of human diseases caused by PTH1R mutations

The Parathyroid hormone/parathyroid hormone-related protein receptor (PTH/PTHrP type 1 receptor; or PTH1R) is a family B G protein-coupled receptor (GPCR) that transmits stimuli provided by two endogenous polypeptide ligands to thereby mediate distinct biological functions (1). PTH1R thus responds to PTH, which is secreted as an endocrine hormone from the parathyroid glands, to maintain homeostasis of calcium and phosphorus and it responds to PTHrP, which is secreted from diverse tissues as a paracrine factor, to play several regulatory roles, most prominently in endochondral bone formation, tooth eruption and mammary gland formation (1–4).

Intracellularly, the classic signaling pathway activated by PTH or PTHrP involves the stimulatory G protein alpha-subunit (Gα_S_) – adenylyl cyclase (AC) – cAMP–protein kinase A (PKA) system. The activated PTH1R can also couple to the Gα_q_ pathway to result in increased IP3/Ca2+ generation (5), as well as recruit βarrestin 1 and 2, key multifunctional effector proteins that classically mediate receptor internalization and desensitization, but can also promote signaling through non-canonical pathways (6).

Most of the inactivating PTH1R mutations found in humans are heterozygous and cause primary failure of tooth eruption (PFTE) (7–12). More than twenty such different mutations are spread throughout the different exons encoding the receptor. All reported PFTE mutations lead to a loss-of-function (LOF) effect, as assessed by direct measurement of signaling *in vitro* or molecular simulations (7–9, 11–13), while at least one intron variant is associated with PFTE (rs1575524795).

Five different heterozygous, gain-of-function PTH1R mutations (H223R, T410P, T410R, I458R, and I458K) have been identified and were shown to cause Jansen’s metaphyseal chondrodysplasia (JMC), a disease characterized by short stature, expanded growth plates and often severe hypercalcemia, hypophosphatemia and hypercalciuria (14–27). These clinical manifestations are consistent with excessive PTH1R signaling in growth plate chondrocytes, bone forming osteoblasts, and kidney target sites (28). All JMC mutations map to one of three amino acid residues in the PTH1R located at the cytosolic base of a transmembrane helices that are involved in a highly conserved network that, when perturbed by the JMC mutations, allows for increased interaction with G protein, and hence agonist-independent receptor activation (29).

**Figure 1:**
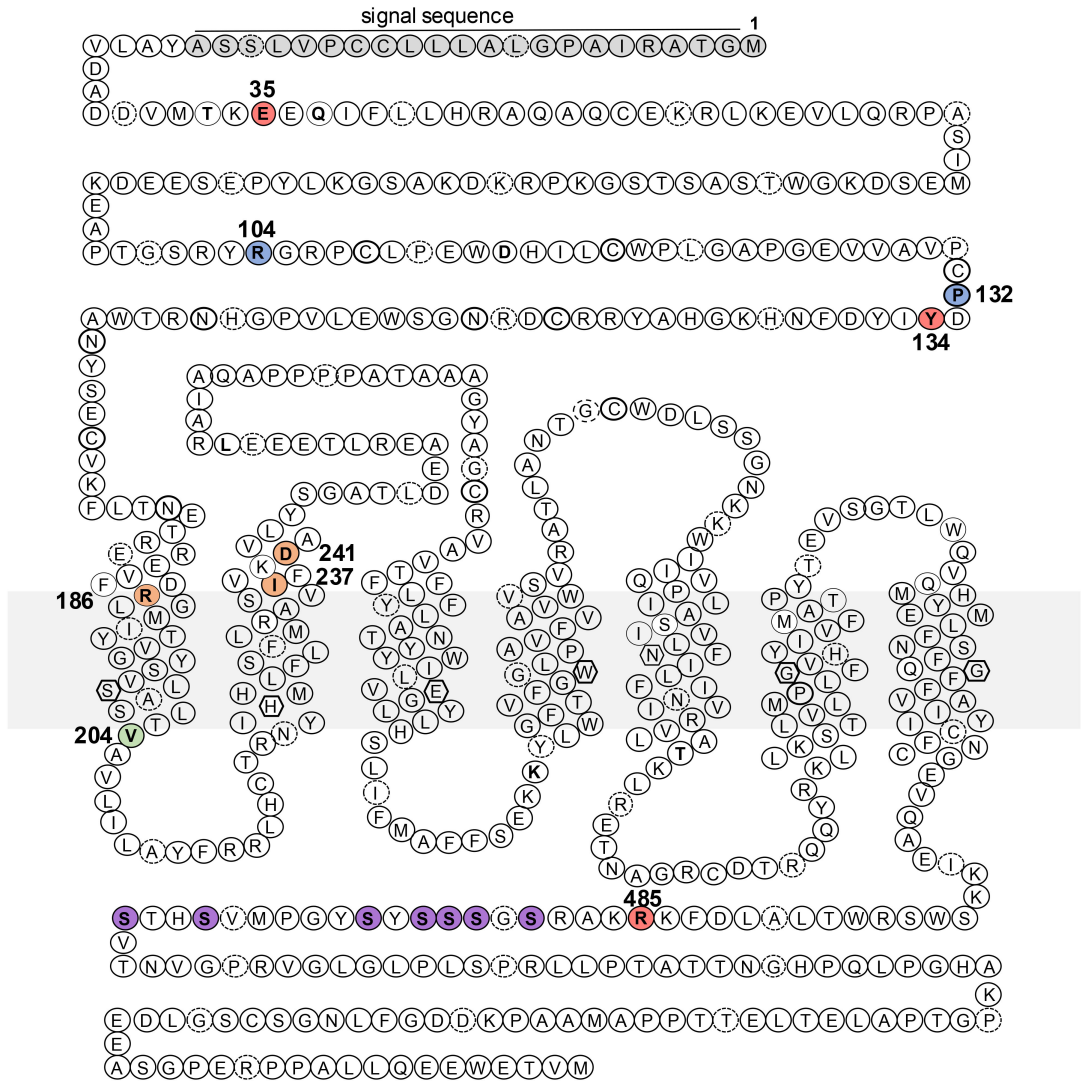
Schematic representation of the PTH1R amino acid sequence and sites of homozygous disease mutations. Indicated in the sequence are the locations of missense or nonsense mutations identified in patients with Blomstrand lethal chondrodysplasia (blue, P132, R104*), Eiken Skeletal dysplasia (salmon, R485, E35, Y132), pseudohypoparathyroidism-like disease (orange, R186, D241, I237) and primary failure of tooth eruption (green, V204). Also indicated are residues of the signal sequence (gray) and phosphorylatable serines (purple) in the C-tail. The gray rectangle represents the plasma membrane.

The consistent impact of heterozygous activating PTH1R mutations in JMC has been well detailed (30, 31), and the heterozygous inactivating mutations in PFTE suggest that both PTH1R alleles are required for normal tooth development (13). In contrast, the twelve reported homozygous PTH1R mutations cause a wider spectrum of clinical abnormalities that are related to disruption of PTH- and/or PTHrP-dependent receptor functions (**Table 1**). Five of these mutations cause severe loss-of-function effects, as seen in Blomstrand’s lethal chondrodysplasia (BLC) (32–43). These mutations cause a profound acceleration in ossification of the entire skeleton, including the rib cage, thus leading impaired lung function and death soon after birth. Seven homozygous non-lethal PTH1R mutations were recently described in patients with various degrees of delayed ossification and PFTE, associated with PTH-resistant hypocalcemia in some cases (44–51) (**Figure 1**). However, these clinical and laboratory findings are distinct from those seen in patients with homozygous PTH mutations that reduce the peptide’s biological activity (52, 53) or heterozygous inactivating mutations in PTHrP that result in brachydactyly and short stature (54–57).

**Table 1:** Summary of clinical syndromes associated with homozygous PTH1R mutants.

Clinical Syndrome	Blomstrand	Failure of tooth eruption	Delayed ossification +/- mild PTH resistance**	Symptomatic PTH resistance +/- delay ossification**
Genotype	P132L, G350fsX351, L373_R383del*, R104X, V365CfsX141	V204E	R485X, E35K, Y134S	R186H, D241E, I237N
Clinical manifestation (PTHrP pathways)	Reduced PTHrP actions in bone and teeth	Reduced PTHrP actions in teeth	Increased PTHrP action in growth plate chondrocytes	Increased PTHrP action in bone for D241E, I237N but not R186H
Clinical manifestation (PTH pathways)	Not studied	No change	Possibly mild reduction in PTH actions	Symptomatic PTH resistance
Main in vitro findings	Severe receptor loss-of-function	Decreased expression	Lack of desensitization to PTHrP signaling	Impaired cAMP response to small/modified PTH analogs

*Compound heterozygous.

**R485X, E35K, Y135S, D241E, I237N described as Eiken syndrome based on the finding of delayed ossification.

The effect of heterozygous disease-causing PTH1R mutants has been reviewed elsewhere (8, 10, 19, 58). We therefore focus our review on homozygous PTH1R mutations (**Table 2**). We first describe the clinical findings in BLC and contrast these findings with those of non-lethal diseases that are caused by homozygous PTH1R mutations. We follow with a pharmacologic characterization of the disease causing PTH1Rs mutants.

**Table 2:** Functional effects of PTH1R mutants.

Disease	Nucleotide change	Protein change	rsID	Allele frequency*	Mutation type	Comment	Main functional effects	Reference
Blomstrand	c.1049+27C>T	p.G350fsX351	rs2107055197	None	Splice donnor	Homozygous	Severe LOF	(32)
Blomstrand	c.1148G>A	p.L373_R383del	rs398122843	0.7	novel splice acceptor site	Compound Heterozygous	Severe LOF	(38)
Blomstrand	c.310C>T	p.R104X	rs121434604	0.14	Nonsense	Homozygous	Severe LOF	(32)
Blomstrand	c.395C>T	p.P132L	rs121434599	0.36	Missense	Homozygous	Severe LOF	(32, 36, 37)
Blomstrand	c.1093delG	p.V365CfsX141	rs1304201852	0.44	Frameshift	Homozygous	Severe LOF	(34)
Eiken (delayed ossification)	c.1453C>T	p.R485X	rs121434603	None	Nonsense	Homozygous	High basal activity and reduced desensitization to PTHrP signaling	(46)
Eiken (delayed ossification)	c.401A>C	p.Y134S			Missense	Homozygous	Reduced desensitization to PTHrP signaling, reduced expression	(49)
Eiken (delayed ossification)	c.103G>A	p.E35K			Missense	Homozygous	High basal activity and reduced desensitization to PTHrP signaling	(51)
PTH resistance and delayed ossification	c.723C>G	p.D241E	rs2107040144	None	Missense	Homozygous	Reduced response to PTH, high basal activity	(45)
PTH resistance and delayed ossification	c.710 T>A	p.I237N			Missense	Homozygous	Reduced response to PTH, high basal activity	(44)
PTH resistance	c.557G>A	p.R186H			Missense	Homozygous	Reduced response to PTH	(48)
Failure of tooth eruption	c.611T>A	p.V204E	rs769180471	0.8	Missense	Homozygous	Reduced response to PTH and decreased expression	(50)

LOF, Loss of function.

*Allele frequency in general population per 100,000. Source: National library of medicine, GnomAD_genomes.

## Clinical syndromes associated with homozygous PTH1R mutations

### Accelerated ossification in Blomstrand lethal chondrodysplasia

During endochondral bone formation, growth plate chondrocytes at the perichondrium synthesize PTHrP to maintain nearby cells expressing PTH1R in a proliferative state and hence delay their differentiation into hypertrophic chondrocytes and the subsequent process of bone mineralization (4, 59).

Mice that are null for PTHrP or the PTH1R die during the neonatal period (60). Their skeleton shows smaller bones in which histology of the growth plates reveals a shortness of the proliferative chondrocyte layer and an overall disruption of the otherwise highly organized zones of differentiating chondrocytes. Neonatal lethality of PTHrP-null mice can be prevented, at least partially, by overexpressing PTHrP transgenically in proliferating growth plate chondrocytes via the collagen type II promoter. These mice, however, show multiple other abnormalities, including altered skin formation, a failure of tooth eruption, and absent mammary glands (61), thus revealing broader roles of PTHrP during development.

The first report of Blomstrand lethal chondrodysplasia (BLC), was presented in 1985 (43). The affected infant was born prematurely at 22 weeks and died shortly after delivery. Postmortem exam showed generalized edema, tongue protrusion due to a short mandible and noticeably short limbs. The only obvious malformation of internal organs was aortic coarctation. Body radiographs showed markedly advanced ossification, which was corroborated histologically by nearly absent proliferating chondrocytes at the end of long bones. Over the next decade several reports followed that were similar in presentation, including fetal hydrops, flat nasal bridge, short limbs and reduced epiphyseal cartilage (32–43, 46). At least two fetuses had cataracts, suggesting hypocalcemia (39). The absence of mammary gland and impact on tooth eruption was documented only later (3).

### Delayed ossification in Eiken skeletal dysplasia

Eiken syndrome was first described in 1984 in three siblings from consanguineous parents (47). In contrast to BLC, all patients appeared normal at birth. When one of the boys was 1 year old, it was noted that his hands and feet were short and broad. Skeletal survey showed delay ossification, which was prominent at the hands and feet. A sibling presented at age 9 years with short stature (3.4 standard deviations below WHO standard) and delayed ossification was noted in the second phalanx of the fifth finger and at the pubic bone, as well as in the epiphyses of other larger bones. The third child was initially evaluated at age 12. At that time, his physical examination revealed no obvious abnormalities, but radiographs revealed a delay in mineralization at the sacrum and metatarsals bones. Subsequent evaluations showed normal ossification in these three patients. Thus, the clinical syndrome described by Eiken et al. is that of delayed ossification.

It was nearly two decades later, in 2005, that Eiken syndrome was ascribed to PTH1R mutations (46). The family described by Eiken was thus found to have a homozygous non-sense mutation at residue arginine 485 (R485X) of the C-terminal tail of the PTH1R.

Two additional homozygous PTH1R mutations, E35K and Y134S, were subsequently described in patients, who presented with delayed ossification (49, 51). The patient with the E35K mutation exhibited delayed ossification as well as supernumerary epiphyses (pseudoepiphyses) and angle-shaped proximal phalanges (51). Interestingly, serum calcium and phosphate levels were within the normal range, but serum PTH was mildly elevated; similar findings of elevated PTH were reported for an additional patient with a homozygous R485X mutation, who was born after the initial description of this family (46). The patient with the Y134S mutation had primary failure of tooth eruption in addition to a delay in growth plate ossification, while serum PTH, calcium and phosphate levels were reported as normal (49).

The bone phenotype in most patients with Eiken syndrome (i.e., delayed ossification) points toward a gain-of-function effect on PTHrP/PTH1R-mediated signaling in the growth plate, as it is well established that this signaling response acts normally to sustain growth plate chondrocyte proliferation and to delay the differentiation of these cells toward the hypertrophic state. Moreover, the clinical syndrome described by Eiken is reminiscent of the findings in transgenic mice that overexpress PTHrP in chondrocytes (62). The other clinical manifestations of Eiken syndrome, however, such as the mild PTH resistance and PFTE, are more likely explained by a loss-of-function effect on PTH1R signaling responses in kidney and teeth, as mediated by PTH and PTHrP, respectively. The identified Eiken mutations thus appear to have pleiotropic effects on PTH1R function that are dependent on the context of cell-type and tissue.

### PTH resistance with or without delayed ossification

Three other independent families have recently been described with delayed ossification, characteristically observed in Eiken syndrome, that was associated with significantly elevated PTH levels, as evidence for PTH-resistance, and symptomatic, in some cases severe hypocalcemia (44, 45). These laboratory abnormalities are reminiscent of pseudohypoparathyroidism, a disorder that is usually caused by impaired G protein expression or function (63). Thus, Demaret et al. reported a boy, who was born at term without obvious skeletal abnormalities. However, by age 1 year, he developed symptomatic hypocalcemia (ionized calcium: 0.68 mM; nl 1.2-1.4), requiring treatment with calcium carbonate and alfa calcidiol. Serum phosphate (3.39 mM; nl 1.36-1.74) and PTH (401 ng/L; nl 14-72) were markedly elevated, which in the context of normal renal function, is consistent with PTH-resistance (45). This patient was found to have a homozygous PTH1R mutation of D241E (c.723C>G). Because of delayed ossification as assessed by hand radiographs, he was diagnosed with Eiken syndrome (45).

More recently, Calder et al. reported two patients with severe hypocalcemia (44). Patient 1 presented at one week of age with hypocalcemic convulsions, while hypocalcemia was not discovered until age 12 years for patient number 2. Both patients had besides hypocalcemia, hyperphosphatemia and elevated PTH levels of 76.4 pmol/L (nl 1.6-7.2) and 36.5 ng/L (nl 1.1-6.9), respectively. In both cases, there were skeletal abnormalities characterized by short metacarpals, extensive metacarpal pseudo-epiphyses and deficient ossification of the sacrum. These patients were found to have a homozygous I237N-PTH1R mutation.

In addition to the above cases classified as Eiken syndrome, a single family from Portugal was identified, in which affected members exhibited PTH resistance but no skeletal abnormalities (48). The index case, a 68-female presented initially at age 22 with epilepsy. At age 49, she came to medical attention with muscle spasms, neurologic deterioration. A head computer tomography revealed calcification of the basal ganglia and cerebellum. During these episodes, she was found to have severe hypocalcemia and hyperphosphatemia, but with normal to mildly elevated serum PTH levels ranging from 45 pg/ml to 72 pg/ml (nl 9-72). The patient had also a blunted urinary cAMP response at 2 and 4 hours after PTH injection. Treatment with calcium and calcitriol improved her hypocalcemia but over the next twenty years her neurologic symptoms progressed, and she became bedridden. This patient was found to have a homozygous PTH1R mutation (R186H), which was also found in two of her siblings, who had elevated PTH levels. Their PTH1R mutation is located near the junction of the extracellular domain (ECD) and transmembrane domain helix-1 (TM1).

### PFTE associated with a homozygous PTH1R mutation

A contrasting phenotype, i.e. skeletal but no biochemical abnormalities, was reported by Jelani et al, who identified 5 members of a five-generation family from Saudi Arabia who presented with PFTE (as in patients with heterozygous LOF PTH1R mutants) (50). While there were no apparent biochemical abnormalities or ossification abnormalities, other variable and minor deformities like palm hyperkeratosis, hypoplastic nasal bridges and clinodactyly were present. These patients were found to have a homozygous V204E missense PTH1R mutation, which maps to the intracellular end of the transmembrane domain helix-2 (TM2) in the PTH1R (64).

## Functional properties of disease causing homozygous PTH1R mutations

### Severe loss-of-function mutations associated with BLC

The first mutation found in BLC affected RNA splicing leading to an 11 amino acid deletion in the receptor (L373_R383del) (38). Despite this large deletion, the L373_R383del PTH1R mutant is well expressed in COS-7 cells but does not bind PTH ligands or mediate ligand-induced generation of cAMP or IP3 (38). Similar loss-of-function effects are seen for other mutants found in patients with BLC (34).

One missense mutation, (P132L) was also described in BLC (37). While cAMP generation is detectable when this mutant is transfected into HEK293 cells and treated with PTH (1–34) or PTHrP (1–36), the overall response is greatly diminished as compared to that seen with the wild-type receptor (65). These findings establish that the BLC phenotype in patients results from a profound reduction in PTH1R signaling.

### Gain of PTHrP function in Eiken syndrome

The homozygous mutations identified in cases of BLC result in severe reductions in PTH1R function when assessed *in vitro*. In contrast, those mutations that are compatible with life have comparatively milder *in vitro* effects in cell-based assays. The R485X truncation, found in Eiken syndrome, results in the loss of most of the PTH1R C-tail, which is important for normal receptor desensitization and the translocation of the receptor from the plasma membrane to endosomes, as mediated by the binding of β-arrestin to the phosphorylated C-tail (66). Consistent with the classical model by which the PTH1R C-tail plays a critical role in recruiting β-arrestin, recent work in transfected HEK293 cells, demonstrated that the R485X mutation strongly impairs the capacity of the PTH1R to recruit β-arrestin2 in response to either PTH or PTHrP ligands (67). As a result, cells transfected with the R485X mutant exhibit increased cAMP generation in response to PTHrP(1–36). Interestingly, the R485X mutant also exhibits increased agonist-independent basal cAMP generation, which could be suppressed by overexpression of β-arrestin2. This result implies that β-arrestin interaction with the PTH1R core region plays a role in the tonic inhibition of signaling by the un-liganded PTH1R (67). These data are further consistent with recent cross-linking studies that identify specific proximity points between β-arrestin2 and both the TM core and C-tail regions of the PTH1R (68, 69).

In contrast to R485X, the E35K and Y134S PTH1R mutations, also found in Eiken syndrome, alter residues in the receptor’s N-terminal ECD. Based on the location of these mutations in the receptor’s ECD, each of the altered amino acids is predicted to affect interaction with the ligand, but not directly with βarrestin, as demonstrated by recent cryo-electron microscopy of the PTH1R in complex with PTH or PTHrP analogs (64, 70–72). In particular, residue 35 of PTH1R are within proximity of residue 19 in PTH and PTHrP (64, 73). Indeed, the two extracellular domain mutations were each found to moderately destabilize the receptor interaction with PTHrP(1–36) but had little if any effect on interaction with PTH(1–34). The decreased binding of PTHrP did not result in detectable reductions in the acute generation of cAMP. However, the mutations did lead to reduced PTHrP-induced βarrestin recruitment and impaired receptor desensitization, which resulted in an increase in cAMP accumulation after repeated stimulation with PTHrP, as compared to that observed on the wild-type receptor under the same conditions (67). The altered effects on PTH vs. PTHrP interaction can be explained by differences between the two PTH1R agonists at key amino acid positions, specifically at position 5 (histidine in PTHrP vs. isoleucine in PTH) and 19 (glutamate in PTH and arginine in PTHrP) (64).

The functional studies thus identified two distinct mechanisms by which Eiken syndrome PTH1R mutations destabilize interaction with βarrestin and hence lead to impaired desensitization of cAMP signaling. On the one hand, the R485X mutation directly removes the C-tail and thus destabilizes the fully engaged PTH1R-arrestin complex. The ECD mutants, E35K and Y134S, impair binding of PTHrP and, as a result, the weaker ligand-receptor complexes do not interact with βarrestin strongly enough to cause receptor desensitization (67). By impairing ligand-induced receptor desensitization, the two ECD mutations thus facilitate a gain-of-function effect on PTH1R signaling. In fact, in the growth plates where local PTHrP levels are expected to be abundant, inefficient PTH1R desensitization is likely to enhance activation of the down-stream signaling pathway resulting in the delayed chondrocyte differentiation that underlies the mineralization defect in Eiken syndrome. It likely would be revealing to generate and study mouse models of Eiken syndrome to test this working hypothesis.

### Mild loss of PTH1R function associated with PTH resistance with or without skeletal abnormalities

Three PTH1R mutations found in patients with symptomatic hypocalcemia and PTH resistance map to the receptor TM1 (R186H) and TM2 (D241E and I237N) (64, 70). The Arginine-186 in TM1 interacts extensively with PTH and PTHrP (residues 3-14) and thus is likely to contribute importantly to ligand binding and activation processes (73, 74). The three PTH1R mutants exhibit surface expression levels that are comparable to those of the wild-type receptor, and their stimulation with either PTH(1–34) or PTHrP(1–36) results in similar cAMP as well as intracellular calcium signaling (44, 65). One important limitation of these *in vitro* assays is that overexpression of the PTH1R in the plasmid-transfected cells can mask subtle changes in ligand-affinity, which nevertheless may be relevant *in vivo* where quantities of the endogenous ligands and receptor are likely much lower. One strategy to probe for more subtle affinity changes involved the use of minimized N-terminal PTH; these peptides retain the capacity to activate the PTH1R but lack the stabilizing interactions which, for intact PTH(1–34)-based ligands, occur between the 15–34 region of the peptide and the N-terminal extracellular domain of the receptor (29, 71). Such N-terminal peptides can thus be more effective at detecting subtle affinity changes caused by mutations that impact the receptor’s ligand-binding pocket (74). Indeed, when probed with modified PTH(1–15) or PTH (1–11) peptide analogs, each of the mutants R186H, D241E and I237N showed a marked reduction in cAMP signaling potency vs. the wild-type receptor (44, 65). For the V204E mutation found in patients with PFTE, the *in vitro* assays revealed a 50% reduction in surface expression levels, which interestingly was also found for the Y134S mutant that also is associated with PFTE. These results with the Y134S and V204E mutants suggest that normal levels of PTH1R expression are required for proper tooth eruption.

## Concluding remarks: unraveling the complex phenotypes of patients with homozygous PTH1R mutations

In contrast to Blomstrand’s lethal chondrodysplasia, several homozygous PTH1R mutations are compatible with life, yet lead to notable and complex phenotypes. Patients with delayed ossification (Eiken syndrome) can exhibit features of both gain-of-function effects of the mutant PTH1R toward PTHrP in developing bone but also loss-of-function effects toward PTH in the kidney (PTH-resistance) or toward PTHrP in developing teeth (PFTE). The functional characterization of these disease-causing mutations revealed varied effects on PTH1R function including diminished desensitization, expression, and subtle yet allele-specific alterations in ligand affinity. The generation of new mouse models should bring even deeper understanding of the cellular and molecular mechanisms causing these diseases, and, importantly, should help advance development of new therapeutic strategies that can be employed more broadly for diseases of bone and mineral metabolism.
